# The roll-out of a health insurance program and its impact on the supply of healthcare services: a new method to evaluate time-varying continuous interventions

**DOI:** 10.1186/s12939-018-0874-1

**Published:** 2018-11-08

**Authors:** Curtis Huffman, Edwin van Gameren

**Affiliations:** 1 Programa Universitario de Estudios del Desarrollo, Universidad Nacional Autónoma de México, National Autonomous University of Mexico, Development Studies Program, Mexico City, Mexico; 2 Center for Economic Studies, El Colegio de México, Carretera Picacho Ajusco No. 20, Ampliación Fuentes del Pedregal, Tlalpan, Mexico City, 14110 Mexico

**Keywords:** Time-varying continuous interventions, Marginal structural models, Inverse probability of treatment weight, Quantile dose-response function, Treatment effect heterogeneity, Mexico, Seguro popular, I38, C21, I13

## Abstract

**Background:**

We analyze the effects of the Mexican universal health insurance program, Seguro Popular, on key variables associated with the provision of healthcare services. Given that the program was introduced gradually over a period that lasted more than a decade, the dynamics of the roll-out of the program and its reaction to the expansion of healthcare services it caused should be accounted for when evaluating the program.

**Methods:**

We present a new semiparametric procedure to analyze time-varying continuous interventions. This is accomplished by bringing together the literatures on continuous and on dynamic treatments. Our approach allows the researcher to estimate mean and quantile dose-response functions by applying local regression methods to appropriately weighted samples that control for time-dependent confounding.

**Results:**

Using administrative data, we show compelling evidence that Seguro Popular has incremented the human and physical resources available for healthcare services over the period 2001–2013. Moreover, we show that these effects have been heterogeneously distributed.

**Conclusions:**

The program has proven most helpful in less vulnerable territories, leaving behind those in greater need.

## Introduction

Traditionally, health insurance in Mexico has been part of the social security system that provides healthcare services, retirement pensions, and several other benefits, for everyone with a formal employer that paid the contributions into the system. Since its introduction in 1943, however, the social security system has remained far away from achieving universal coverage. Basically, all informal workers and their families, about half of the population, remained uncovered and therefore had very limited access to affordable healthcare services. At the start of the millennium, the newly-installed government proposed and enacted a health insurance program for those in informal employment, with the intention to achieve universal health coverage [1, 2]. By 2014, almost everyone previously uninsured, about 55 million Mexicans, had obtained health coverage via the new public insurance system (SP) that had been introduced next to the (already fragmented) social security system. The program’s funding, largely through transfers from the federal government to the states based on the number of affiliates in each state, goes without strict earmarking. Hence, the expansion of healthcare services was not automatically implied; States had a relatively large autonomy to decide how to spend the additional resources. Therefore, whether the financial resources managed to translate into the provision of healthcare services (potentially) demanded by the newly-insured is an important matter that deserves attention.

We intend to estimate the effect of the health insurance program, (SP), on key variables associated with the provision of healthcare services in Mexico. An important characteristic of the program is that it unfolded with varied intensity over a period of more than 10 years. Therefore, analyzing its effects under the assumption of a single homogeneous action at a single point in time is far from reality. When a program evolves over time in a deterministic way, it may be possible to control for confounders using only preprogram covariates (see for example [3]). If, on the other hand, the effectiveness of the intervention at earlier stages of implementation were to affect its nature at subsequent stages, the (non-deterministic) dynamics of the intervention itself generates potential confounders, which are impossible to control for before the program takes place^1^. Given that the roll-out of SP did not follow a predetermined plan, the dynamics should be accounted for when evaluating the program.

Robins et al. [4], RHB from now on, presented a set of tools to estimate the causal effects of dynamic processes from Time-series Cross-Sectional (TSCS) data –repeated measurements of the same units at several points in time. These tools explicitly model the dynamic selection inherent in these time-varying processes, overcoming the biases derived from wrongly assuming a single-shot kind of intervention. RHB’s methodology applies Inverse Probability of Treatment Weights (IPTW) to a class of semiparametric models called Marginal Structural Models (MSMs). In these MSMs, each unit is weighted by the inverse probability of its observed intervention history, creating a pseudo-sample where dynamic selection is eliminated. Then, using this pseudo-sample, the MSM dictates the form of the relationship between the histories and the outcome of interest. In the MSM literature the intervention histories are typically coded as sequences of a binary variable; that is, histories of interventions that either take place or not at every point in time [5], thus leaving out the case of interventions that, at every stage, may occur with varied intensity – best coded as a continuous variable– such as the SP program under scrutiny in this paper.

We propose a procedure that takes the application of RHB’s methodology for binary dynamic processes to an implementation for continuous treatments. We use the resulting IPTWs to estimate Mean and Quantile Dose-Response Functions (DRFs) in these dynamic contexts, analyzing the impact SP has had on the expansion of the number of doctors’ offices and of physicians and nurses with day-to-day contact with patients.

In line with previous research [6, 7], we found that SP has had a positive effect on the infrastructure and human resources of the Secretaría de Salud –Ministry of Health– (SSA). However, the methodology implemented here allowed us to address a richer set of causal questions as we are now in position to analyze in detail the response to the gradual roll-out of the program. Because of this expanded scope, we also found evidence that the program has had heterogeneous effects across the regional administrative units of the SSA, exhibiting greater impacts on those units with greater densities of human and material resources, thus leaving behind those units with scarcer resources. In comparison with previous research, considering the dynamic nature of the treatment deflates the impact of the program by approximately one third.

The paper proceeds as follows. “Causal inference with time-varying continuous interventions” section describes how RHB’s methodology has extended the methods focused on the effect of a single homogeneous action at a single point in time, followed by the introduction of our extension of these estimation procedures to time-varying continuous interventions. In particular, it describes the weighting approach that permits the estimation of dynamic mean and quantile dose-response functions. “The effect of Seguro popular on the supply of healthcare services” section applies the techniques in the estimation of the effects of the SP program on the supply of healthcare services. “Conclusions” section concludes with some public policy implications of the analysis.

## Causal inference with time-varying continuous interventions

Although the risk that the observed correlation between the outcomes and the treatment is due to some other confounding variable is not different from single homogeneous interventions, in dynamic settings such as the one under scrutiny in this paper, confounders may share the time-varying nature of the intervention. A time-varying confounder can, potentially, both affect future exposure to the program as well as be affected by past exposure [8]. In this section we briefly review how the impact of time-varying bivariate treatment exposures –with confounders that may both react to as well as cause the changes in the environment– has been analyzed in the literature, and we address how we can implement an extension to continuous treatment exposures that will allow us to evaluate the program at hand.

### Notation and main assumptions

By way of a generalization of IPTW estimators [9] for longitudinal studies, RHB presented a framework to estimate the effects of dynamic interventions in the presence of time-varying confounders from TSCS data. Like other impact evaluation methods based on observational data, RHB’s approach relies on the ability to identify and measure all possible confounders, in this case repeatedly over time; that is, on a dynamic version of the well-known “selection on observables” or “ignorability” assumption.

In the counterfactual framework of dynamic causal inference, this assumption is referred to as sequential ignorability [8] or conditional interchangeability [10], and states that the exposure level at any point in time is statistically independent of the potential outcomes, conditional on the covariate and exposure histories up to that point. In statistical terms, let *i* index program recipients, with *i*=1,…,*n*, and let *t*=1,…,*T* be the stage of the intervention, where *T* is the last stage of the program recorded in the data. At every stage *t*, recipients are observed receiving the exposure level *a*_*it*_ to the benefits of the program; that is, one possible realization of the exposure variable *A*_*t*_ 2. Collecting the observed exposures to the program for a given recipient up to stage *t* gives us the history of exposure, $\underline {a}_{it}=(a_{i1},\ldots,a_{it})$, an occurrence of the collection of possible exposures up to *t*, $\underline {A}_{t}=(A_{1},\text {\dots },A_{t})$. Let $\underline {\mathbf {X}}_{t}$ and $\underline {x}_{it}$ be similarly defined for a covariate history, where **X**_*t*_ is the most recent set of variables that could have affected *A*_*t*_, and that may have been affected by past exposure, $\underline {A}_{t-1}$.

Each possible exposure history $\underline {a}$ has an associated potential outcome $Y^{\underline {a}}$. Naturally, any recipient of the program exhibits only one of these potential outcomes, $Y_{i}=Y_{i}^{\underline {a}}$, the one associated with its own particular exposure history $\underline {A}_{i}=\underline {a}$. As is common in causal inference, we assume that a recipient’s exposure history does not depend on the exposure histories of other recipients –the stable unit treatment value assumption. Taking together all recipients and all realizations of $\underline {a}$, the collection of observed potential outcomes, $Y|\underline {A}$, is equal to the associated potential outcome, $Y^{\underline {A}}|\underline {A}=Y|\underline {A}$. The other potential outcomes, the ones that were not observed because they did not actually occur, are said to be counterfactual.

With this notation, the assumption of sequential ignorability states that for any exposure history $\underline {a}$, the potential outcome associated with that exposure history, $Y^{\underline {a}}$, is independent of the exposure history up to stage *t* given the covariate and exposure histories up to that stage, that is, $Y^{\underline {a}}\bot A_{t}| \underline {\mathbf {X}}_{t},\underline {A}_{t-1}=\underline {a}_{t-1}$. It is also important to note that to compare the various exposure histories, IPTW estimators depend on assuming, firstly, that one has a consistent model for the probability density function (pdf) of *A*_*t*_ given $\underline {A}_{t-1}$ and $\underline {\mathbf {X}}_{t}$ (that is, the probability of *A*_*t*_) and, secondly, that at any stage *t*, it is not the case that there is a covariate history $\underline {x}_{t}$ and past exposure $\underline {A}_{t-1}$ such that all recipients with such histories are certain to receive the identical exposure *a*_*t*_. That is, if $f_{\underline {A}_{t-1},\underline {\mathbf {X}}_{t}}\left [\underline {a}_{t-1},\underline {x}_{t}\right ]\neq 0$, then $f_{A_{t}|\underline {A}_{t-1},\underline {\mathbf {X}}_{t}}\left [a_{t}|\underline {a}_{t-1},\underline {x}_{t}\right ]>0$ for all *a*_*t*_ – each exposure history must have a positive probability of occurring. This last assumption is closely related to the traditional of common support assumption; in “Covariate balance and common support” section we address how this issue is approached in a dynamic continuous setting.

### Inverse probability of treatment weights

The problem researchers face in estimating the causal effect of a program is that typically the potential outcomes, $Y^{\underline {a}}$, do not have the same distribution as the observed outcomes, $Y|\underline {A}=\underline {a}$; that is, $f_{Y^{\underline {a}}}\neq f_{Y|\underline {A}=\underline {a}}$, or equivalently $f_{Y^{\underline {A}}}\neq f_{Y|\underline {A}}$. This (general) problem is exacerbated in the dynamic context at hand, because with time-varying confounders, particular levels of exposure followed recipients with particular responses to the program, which makes recipients incomparable.

However, what RHB showed is that, under the aforementioned assumptions (sequential ignorability, consistency and positivity), it is possible to generate a reweighted version of the observed outcomes that, conditional on $\underline {a}$, have the same distribution as the potential outcomes. All that is needed is a consistent model for the pdf of *A*_*t*_ given $\underline {A}_{t-1}$ and $\underline {\mathbf {X}}_{t}$ – informally, for the probability of observing the exposure history that recipient *i* actually experienced.

If we assume that all confounders are observable, the idea is to decompose the joint distribution of the observed data into an equivalent product of conditional probabilities representing different causal mechanisms. Doing this allows us to find the weighting function that removes all the factors from the product that are in excess to simulate the potential outcome distribution.

To illustrate the mechanism, assume that we have two covariates, *X*_1_ and *X*_2_, two stages with exposures, *A*_1_ and *A*_2_ and one outcome, *Y*. Assume further that the temporal order of these variables is (*X*_1_,*A*_1_,*X*_2_,*A*_2_,*Y*) and that every variable is affected by all its predecessors. In this case, we would have that the joint pdf of the observed values of *Y*, *A* and *X* is:

1 $$ {\begin{aligned} f_{Y|A_{1},A_{2}} & = f_{Y^{\underline{A}}|A_{1},A_{2}} = \frac{f_{Y^{\underline{A}},A_{1},A_{2}}}{f_{A_{1},A_{2}}} = \frac{\int \int f_{Y^{\underline{A}},A_{1},A_{2},X_{1},X_{2}}dx_{1}dx_{2}}{f_{A_{1},A_{2}}}\\ &=\! \frac{\int \int f_{A_{2}|X_{1},A_{1},X_{2},Y^{\underline{A}}}*f_{X_{2}|X_{1},A_{1},Y^{\underline{A}}}*f_{A_{1}|X_{1},Y^{\underline{A}}}*f_{X_{1}|Y^{\underline{A}}}*f_{Y^{\underline{A}}}dx_{1}dx_{2}}{f_{A_{2}|A_{1}}*f_{A_{1}}}\\ \qquad &=\frac{\int \int f_{A_{2}|X_{1},A_{1},X_{2}}*f_{X_{2}|X_{1},A_{1},Y^{\underline{A}}}*f_{A_{1}|X_{1}}*f_{X_{1}|Y^{\underline{A}}}*f_{Y^{\underline{A}}}{dx}_{1}{dx}_{2}}{f_{A_{2}|A_{1}}*f_{A_{1}}}. \end{aligned}}  $$

where the first equality follows from consistency, the second and fourth from the definition of a conditional pdf, the third from the definition of a marginal pdf and the last from sequential ignorability. At this point the reweighting function suggests itself as

2 $$ SW(\underline{A},\underline{X})=\frac{f_{A_{2}|A_{1}}*f_{A_{1}}}{f_{A_{2}|X_{1},A_{1},X_{2}}*f_{A_{1}|X_{1}}}.  $$

Applying these weights to the distribution of the observed data enables the simulation of the distribution of $\phantom {\dot {i}\!}f_{Y^{A}}$:

3 $$ {}f_{Y^{\underline{A\!}}}\,=\,SW\!(\underline{A},\underline{X})*f_{Y|A_{1},A_{2}}\,=\,\!\!\int \!\!\!\!\int \!\!f_{X_{2}|X_{1},A_{1},Y^{\underline{A}}}*f_{\!X_{1}|Y^{\underline{A}}}*f_{\!Y^{\underline{A}}}{dx}_{1}{dx}_{2}.  $$

The use of the transformed data allows us to estimate the causal effects of the program. Intuitively, as the weighting replaces *f*_*A*|*X*_ with *f*_*A*_ in the observed data, the process alters the distribution of *A* breaking the links between the exposure and the factors that affect it. With longer histories, this leads naturally to the stabilized weights proposed by RHB as

4 $$ SW(\underline{A},\underline{\mathbf{X}})=\prod\limits_{t=1}^{T}\frac{f_{A_{t}|\underline{A}_{t-1}}}{f_{A_{t}|\underline{A}_{t-1},\underline{\mathbf{X}}_{t}}}.  $$

Notice that in the denominator of the weight corresponding to recipient *i*, *S* *W*_*i*_, we find the probability density of the observed exposure history of that recipient conditional on the past, expressed as the product of the respective probability density at every stage, hence the name Inverse Probability of Treatment Weighting. As we have seen, the *SW* removes any confounding by ensuring that the distribution of exposure histories $\underline {a}$ is not related to the measured confounders $\underline {\mathbf {X}}$. In this way, confounders cannot be accountable for any remaining differences between exposure histories. Given that, in the reweighted data, the connection between the exposure histories and the confounders has been broken, one can simply run whatever model one would have used in the case of a randomized evaluation. Of course, in nonrandomized evaluations, the weights $SW(\underline {A},\underline {\mathbf {X}})$ are unknown, and have to be estimated.

### Estimating the stabilized weights

To estimate the *SW* we need to model the exposure in each stage, conditional on the past. When exposure to the intervention comes as a single action that either occurs or not, a common approach is to estimate the probability of being exposed, $p(A_{t}=1|\underline {A}_{t-1},\underline {\mathbf {X}}_{t})$, with a standard logistic regression model. This has been known in the literature as the *propensity score*.

In the case of continuous interventions, RHB suggest the use of Ordinary Least Squares (OLS) regression of *A*_*t*_ on $\underline {A}_{t-1}$ and $\underline {x}_{t}$ to model the distribution of the observed exposure given the past, i.e., $A_{t}|\underline {A}_{t-1},\underline {X}_{t} \boldsymbol {\sim } N\left (h(\underline {A}_{t-1},\underline {\mathbf {X}}_{t};{\boldsymbol {\alpha }}),\sigma ^{2}\right)$. For instance, we might have $h(\underline {A}_{t-1}, \underline {\mathbf {X}}_{t}; {\boldsymbol {\alpha }}) =\alpha _{0}+{\boldsymbol {\alpha }}_{1} \underline {A}_{t-1}+ {\boldsymbol {\alpha }}_{2}\underline {\mathbf {X}}_{t}$, which models exposure as a linear function of the whole treatment and covariate histories up to the current stage. An estimate of the weights requires an estimate of the parameter vector (***α***,*σ* ^2^). We can obtain these estimates, $\left (\hat {\mathit {\alpha }}_{0},\hat {\boldsymbol {\alpha }}_{1},{\hat {\mathit {\boldsymbol {\alpha }}}}_{2},\hat {\sigma }^{2}\right)$, from a pooled OLS, treating each recipient-stage as a separate observation. This would amount to use the normal distribution as the basis for the estimation of the denominator of the weights,

5 $$ \hat{f}_{A_{t}|\underline{A}_{t-1},\underline{X}_{t}}=\frac{1}{\hat{\sigma}\sqrt{2\pi}}e^{-\frac{1}{2}\left\{ \frac{a_{t}-(\hat{\alpha}_{0}+\boldsymbol{\hat{\alpha}}_{1}\underline{A}_{t-1}+\hat{\boldsymbol{\alpha}}_{2}\underline{\mathbf{X}}_{t})}{\hat{\sigma}}\right\}^{2}},  $$

while for the numerator, $\hat {f}_{A_{t}|\underline {A}_{t-1}}$, all that is required is an additional model without conditioning on the time-varying covariates. Note that there is no need to assume a Gaussian distribution; in principle, one can estimate $f_{A_{t}|\underline {A}_{t-1},\mathbf {\underline {X}}_{t}}$ as the pdf of the exposure assignment model, evaluated at the observed exposure and covariates histories for the distribution and function *h*, that better suits the program under analysis. Hirano and Imbens [11] coined the term Generalized Propensity Score (GPS) for the cross-sectional version of this quantity, because it is analogous to the propensity score for binary coded exposures. Once we have obtained $\hat {f}_{A_{t}|\underline {A}_{t-1},\mathbf {\underline {X}}_{t}}$ and $\hat {f}_{A_{t}|\underline {A}_{t-1}}$ for every recipient-stage, all we need to construct the weights is to take the products and divisions across stages to obtain the estimates $\widehat {SW}(\underline {A},\underline {X})$.

However, RHB’s suggestion does not take you all the way as to how to actually implement this continuous extension, leaving out important details which complicate matters in this context. In the remainder of the paper we work out these details, for the first time, and apply them to the evaluation of the SP program.

### Covariate balance and common support

In the single-shot framework a crucial diagnostic to validate the estimated propensity score consists of checking for covariate balance. We have seen in “Inverse probability of treatment weights” section that under the assumptions of sequential ignorability, consistency and positivity, the exposure is unconfounded in the weighted data, conditional on past exposure. That is, once we have broken the link between the exposure and the covariates, at any given stage of the program, the exposure might differ on a time-varying confounder, but these differences will have to do with past exposure. This implication led Blackwell [8] to suggest a balance test in a time-varying context.

If after reweighting the data and conditioning on past exposure, $\underline {A}_{t-1}$, *A*_*t*_ is still predictive of *X*_*t*_, then it is likely that we have residual confounding of the relationship between the outcome and the exposure [8]. We can check for these associations comparing an unweighted and weighted pooled regression of each covariate *X* at stage *t* on the cumulated exposure before *t*, and the level of exposure at *t*. We would expect that the coefficient associated with the latter regressor, *A*_*t*_, would be statistically insignificant in the weighted data.

Also of importance in the impact evaluation literature are “common support” or “overlap region” considerations. In the single-shot framework these terms refer to restrict the estimation of causal effects to comparable recipients: those with similar distributions of covariates across exposures. The idea is to avoid the bias that may arise when the support of the distribution of **X** differs among groups with different degrees of exposures. In the case of fixed continuous interventions, a natural strategy, proposed by Flores et al. [12] – FFGN from now on–, to check for common support is to split the range of the exposure in blocks and compare the distribution of the covariates among all of them.

As the number of blocks grows and ultimately reaches the number of observations in the data, comparisons end up being on individual recipients and how “far away” every one of them is from the rest in terms of the covariates. The farther away recipient *i* is from the rest, the less comparable it is, since its particular value of **X**_*i*_ sets it apart from the rest of the recipients. Intuitively, it differs too much from the other recipients in the data and so is not readily useful for estimating causal effects.

King and Zeng [13, 14] have proposed the use of Gower [15, 16]’s metric as the basis for the assessment of the distance between one point and the rest of the data, since this measure is appropriate for both continuous and discrete variables. The Gower distance between two points *x*_*i*_ and *x*_*j*_ is defined as the average absolute distance between the elements of the two points, divided by the range of the data:

6 $$ G_{ij}^{^{2}}=\frac{1}{K}\sum\limits_{k=1}^{K}\frac{|x_{ik}-x_{jk}|}{r_{k}},  $$

where the range is *r*_*k*_=*m* *a* *x*(*X*_*k*_)−*m* *i* *n*(*X*_*k*_) and the *min* and *max* functions return the smallest and largest elements, respectively, in the set including the *k*th element of the covariates **X**. Informally, we can see *G* ^2^ as the distance between two points expressed as a proportion of the distance across the data – although technically speaking the distance measure is *G*, the square root of this quantity.

A natural possibility is to use the mean distance between the recipient under scrutiny and the rest of the observations. In this fashion a mean value of $G_{ij}^{^{2}}$ over all *j*s equal to 0.5 means that the average distance between recipient *i* and all other recipients requires to travel the equivalent distance as 50% of the way across the data set. Nonetheless, in a time-varying context – with recipient-stage observations– defining the cut-off value is not sufficient to determine which observations to compare. Given the progressive way the weights $\widehat {SW}(\underline {A},\underline {\mathbf {X}})$ are estimated, restrictions on the data should be imposed sequentially, having eliminated recipients that are found to be too far away from the rest in terms of **X**_*t*_ before analyzing **X**_*t*+1_, keeping in the end only those recipients whose covariates **X**_*t*_ are deemed “nearby” at every stage *t*.

It is important to note that removing the recipients outside common support from the analysis changes the population of inference, restricting it, in principle, to those recipients for which we can produce reliable answers to our causal questions. Once the common support has been determined, the exposure assignment model estimated and the stabilized weights validated by checking for covariate balance, we can use them to estimate the causal quantities of interest.

### Mean and quantile dose-response functions for time-varying continuous interventions

With time-varying interventions, estimating the causal effect of every exposure history as we would in a single-shot framework, proves a technically impossible task because even with interventions that only take a handful of different degrees of intensity at every stage, the number of possible exposure histories grows geometrically with the number of stages, let alone with a continuum of different degrees of exposure.

To estimate the effect of a time-varying intervention we need additional assumptions about which exposure histories should have similar potential outcomes: a response model that would allow for the entire exposure history to affect the outcome in a structured, low-dimensional way. We may assume, for instance, that recipients with the same cumulative exposure should have similar potential outcomes.

The substance of the evaluation, and more often than not the amount of data on hand, will determine what response model makes sense for the potential outcome. If summarizing the recipients’ exposure histories with a scalar function, such as the cumulative exposure $cum(\underline {A}_{ik})=\sum _{k=1}^{t}a_{ik}$, happens to be reasonable, we could avoid making additional assumptions about the functional form of the response model using nonparametric regression methods, a strategy first suggested by FFGN in the context of fixed continuous interventions^3^. In this way, within a local linear regression framework, we could estimate a mean response curve that maps the mean effects of the program in relation to the cumulative exposure to it: a mean Dose-Response Function (DRF) for the cumulative exposure.

Since the pseudo-sample generated by $\widehat {SW}(\underline {A},\underline {\mathbf {X}})$ simulates the entire distribution of the potential outcome, it is also possible to use the same kernel smoothing methods, this time with quantile regression, to examine not just the mean, but any quantile effects of the cumulative exposure to the program and address its potential distributional effects on the outcome. In other words, we can also use the same nonparametric regression methods to estimate any quantile DRFs for the cumulative exposure.

## The effect of Seguro popular on the supply of healthcare services

In this Section, the procedures so far discussed are used to estimate the effects of the Mexican universal health insurance program, Seguro Popular (SP), on the actual capacity of the Secretaría de Salud –Ministry of Health– (SSA) to meet the new demand for healthcare services. In particular, as outcome variables we consider the observed increment in the relative number (per 1000 people outside the social security network) of SSA’s doctors’ offices, physicians and nurses in day-to-day contact with patients (providing clinical care), which can be considered the most basic human and material resources involved in the provision of healthcare services.

We use administrative records of the SP program containing the number of families and individuals affiliated from 2002 to 2013, and the federal records on infrastructure and human resources employed by the SSA over the same time period^4^. Information regarding the quality of the healthcare services, for example on the precise qualifications of the physicians and nurses, or on waiting lists, is not available, although more recently pro gress is being made on the recording of quality indicators^5^.

**Fig. 1:**
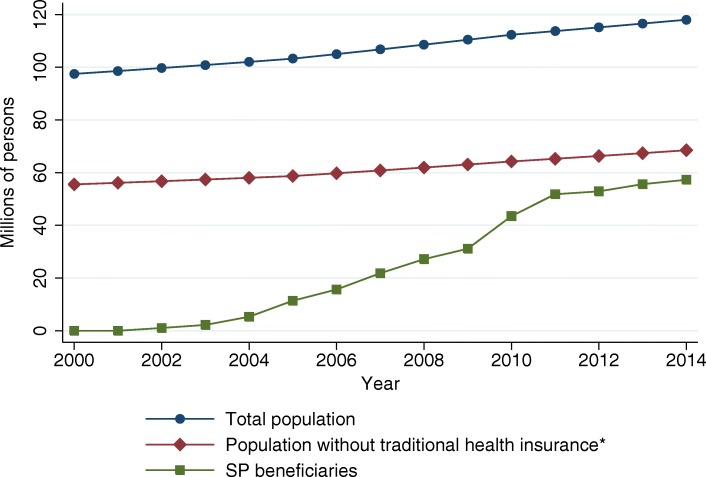
Seguro Popular roll-out. *Traditional health insurance refers to the insurance provided by social security institutions such as IMSS, ISSSTE, Pemex, the Ministry of Defense or the Navy to all formal workers. Source: Own elaboration based on data from the Census on Population and Housing and administrative records from Seguro Popular

Enrollment into the system is voluntary and is granted to all legal residents in Mexico who lack health insurance, ascertained with the mere declaration of the applicant. Figure 1 shows the evolution of the roll-out; just over 10 years after the initial pilots the SP covers more than 55 million people.

Although SP was meant to correct previous imbalances in the distribution of financial, physical, and human resources across the federal states [17], its implementation was a process that unfolded over time at a non-deterministic pace that was different from place to place. The financial resources were primarily obtained through a social contribution to the states, covered annually by the federal government directly proportional to the number of beneficiaries in each state. These resources were intended for the expansion of healthcare services but came without strict earmarking. The states had the responsibility for the assignment of resources to the Sanitary Jurisdictions (SJs), the regional administrative units accountable for the provision of healthcare services, but they were relatively autonomous in deciding about the preferred way of spending^6^. Misalignment of incentives between state-level institutions is also pointed out as a main obstacle in broadening the supply of healthcare services while maintaining previously existing regional disparities [17]. Despite these issues, former and current Health Ministers corroborate that SP has been the spearhead program through which the Mexican Health Ministry has been channeling the financial resources to meet the healthcare needs of the population not covered by the social security in Mexico [17].

The SP program is therefore a clear example of a time-varying intervention. Moreover, it is not unlikely that the roll-out of the program was affected by the density of SSA’s personnel and infrastructure in its regional administrative units and that, at the same time, the program helped build up these human and material resources. In such a situation, it becomes necessary to account for this dynamic selection if one is to assess the effectiveness of the program, for which, moreover, addressing regional disparities is suggested to be an important theme.

### Evaluations of Seguro popular

The existing evaluations have focused on households’ healthcare expenditures, use of services, and labor market effects, without taking into consideration the potential relevance of the gradual roll-out of the program. Key findings include that SP affiliates see reduced their out-of-pocket health expenditures [18–25] and catastrophic health expenses [26]. Increments in the use of services have also been reported [18, 19, 23, 27], as well as positive effects not just on the self-reported health of the beneficiaries [18], but also in some biomarkers [23]. Another strand of the evaluation literature of SP has focused on its impact on the labor market, as the program might be creating perverse incentives to work in the informal sector. However, the evidence is mixed. Some studies find negligible or no effects [28–31], while others find that the SP has the hypothesized negative effect on the creation of formal jobs, especially in small and medium sized firms [6].

Practically no attention has been paid to the effects that the expansion of the SP program might have had on the supply side of the healthcare services. So far, only Bosch and Campos-Vázquez [6] have tackled this issue, providing evidence that SP has had a positive impact on the number of physicians, nurses and clinics in Mexican municipalities. They followed a difference-in-difference approach conceptualizing the implementation of the SP program as an event that occurs in a municipality once more than 10 individuals have been affiliated, hence, ignoring both the duration of the roll-out process and the different levels of treatment intensities observed during that process. Huffman and van Gameren [7] also report positive but heterogeneous impacts of SP on the increment of resources, using a continuous treatment indicator. However, they ignore the time-varying aspects of the roll-out of SP.

### Common support and covariate balance tests

In our analysis we focus on the 233 SJs as recipients of SP coverage, defined as the number of persons affiliated to the program relative to the size of the target population in every SJ, that is, the number of individuals who do not have access to the health insurance provided by law through the social security system to formal workers^7^. In Mexico, SJs are the basic administrative units of the SSA, and they are the bodies in charge of the operation of healthcare services and its programs, and therefore the natural unit of analysis. Note that the SP coverage attained by SJs at any given year is the result of a gradual nonstop affiliation effort that started in 2002; that is, a history of increments in the exposure to the program registered yearly in our data from 2002 to 2013.

If we were to estimate the effect of SP coverage as we would in a randomized evaluation, that is, as if we knew that $Y^{\underline {a}}\perp \underline {A}$, we may well induce bias in the causal estimates. This can occur because it is likely that there are characteristics of the SJs that not only may have influenced the exposure history to the program ($\underline {a}_{i}$) but that are also related to the SJs’ capacity to invest in human and material resources (*Y*_*i*_). It is natural to assume that SJs that exhibit a lower density of human and material resources, for one reason or another, experience difficulties to translate the financial resources into additional investments in personnel and infrastructure. If this lower density of resources had been a criterion for intensifying the coverage efforts of SP, or even if in a more mechanical way the pace of the affiliation efforts were determined by the availability of SSA’s personnel, then particular exposure histories would have followed SJs according to their capacity to respond to the program, thus biasing naive estimates.

To address these concerns, we include in our estimates of SP’s effect the density of resources employed by the SSA in the provision of healthcare services as time-varying covariates ($\underline {\mathbf {X}}_{i}$): doctors’ offices, staffed and non-staffed hospital beds, physicians with and without day-to-day contact with patients and nurses with and without day-to-day contact with patients^8^. For these 7 variables we have determined the common support using Gower’s measure as described in “Covariate balance and common support” section, keeping only those SJs whose average distance to the rest was less than 0.5 at every stage. Following this rule, we have kept 194 SJs, a balanced panel of 2328 SJ–year observations, discarding the 17% least comparable SJs and making our estimates less model-dependent [14].

Tables 2 and 3 in “Appendix” section report means and standard deviations before and after discarding the least comparable observations. After trimming the sample, the data is more compact in every dimension, making statistical fitting more precise and, therefore, our estimates more reliable. It is worth noting that, according to the density of resources, the SJs dropped from the sample belong mostly to the upper part of the distribution (with more resources). Also, the within decomposition shows that the trimmed part of the sample exhibits the greater variability, mostly upward but also downward.

Regarding our exposure assignment model to estimate RHB’s stabilized weights (“Inverse probability of treatment weights” section), since the observed change in the coverage of SP, *A*_*t*_, is a continuous variable, we estimate $\hat {f}_{A_{t}|\underline {A}_{t-1},\underline {\mathbf {X}}_{t}}$ from a pooled OLS on all 2328 SJ-year observations in the trimmed sample, using the 7 time-varying covariates described above, all their two-way interactions, as well as past SP coverage, i.e. $cum(\underline {A}_{t-1})$, as regressors. Likewise, for $\hat {f}_{A_{t}|\underline {A}_{t-1}}$, we use the same model using only $cum(\underline {A}_{t-1})$ as regressor.

After constructing $\widehat {SW}(\underline {A},\underline {\mathbf {X}})$, we follow Blackwell [8] and check for residual confounding, comparing an unweighted and weighted pooled regression of each of the 7 covariates on the past SP coverage, $cum(\underline {A}_{t-1})$, and the increment in the coverage at *t*, *A*_*t*_ (“Covariate balance and common support” section). Table 1 shows the results of this balance test. There we see that the coefficient associated to the change in SP coverage, *A*_*t*_, turns statistically insignificant in the weighted data – the pseudo-sample generated by $\widehat {SW}(\underline {A},\underline {\mathbf {X}})$’ indicating that weighting restores unconfoundedness and that causal interpretations are permitted.

**Table 1:** The Change in History-Adjusted Balance between the Weighted and Unweighted Data^a^

Variable		*t*-statistic of *A*_*t*_	*p*>|*t*|
Doctors’ offices	Unweighted	2.59	0.010
	Weighted	0.76	0.447
Staffed hospital beds	Unweighted	0.03	0.975
	Weighted	0.41	0.679
Non-staffed hospital beds	Unweighted	2.60	0.009
	Weighted	0.07	0.944
Physicians in day-to-day	Unweighted	3.46	0.001
contact with patients	Weighted	1.20	0.228
Physicians without day-to-day	Unweighted	-0.69	0.491
contact with patients	Weighted	0.01	0.990
Nurses in day-to-day	Unweighted	3.01	0.003
contact with patients	Weighted	0.92	0.358
Nurses without day-to-day	Unweighted	-1.71	0.087
contact with patients	Weighted	-0.23	0.820

Source: Own elaboration based on data from the National Health Information System (SINAIS) and the administrative records of Seguro Popular

^a^These estimates come from pooled regressions of the time-varying covariate at year *t* on SP coverage before year *t* and the change in SP coverage observed in year *t*

**Fig. 2:**
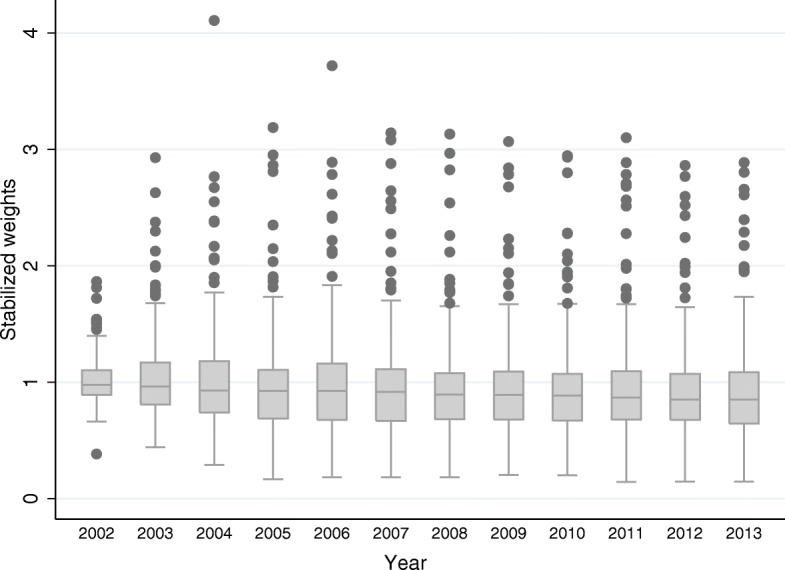
Stabilized Weights Over the Years. Source: Own elaboration based on data from the National Health Information System (SINAIS) and the administrative records of Seguro Popular. The boxes are the yearly inter-quartile ranges and the horizontal line inside of each box corresponds to the median. Whiskers represent the smallest observation still within 1.5 Inter Quartile Range of the lower quartile, and the largest observation still within 1.5 Inter Quartile Range of the upper quartile. Outliers which appear as dots

Cole and Hernán [5] noted that a lack of common support tends to push weights away from 1. Intuitively, it would correspond to cases where the probabilities of certain exposure histories are close to 0 or 1 in some parts of the covariate space, which can be interpreted as a violation of positivity. In Fig. 2 we show the final distributions of the stabilized weights by year *t*,x

7 $$ \widehat{SW}_{t}(\underline{A}_{t},\underline{\mathbf{X}}_{t})=\prod_{k=1}^{t}\frac{\hat{f}_{A_{k}|\underline{A}_{k-1}}}{\hat{f}_{A_{k}|\underline{A}_{k-1},\underline{\mathbf{X}}_{k}}},  $$

where we truncate the product up until the corresponding year. We see that the means at each year are close to 1, and that the minimum and maximum values are also reasonably close, indicating well-behaved weights.

### The effects of Seguro popular: mean dose-response functions

We are interested in estimating a response model for SP coverage, in other words, for the cumulative exposure history $cum\left (\underline {A}_{k}\right)={\sum \nolimits }_{k=1}^{t}a_{k}$. Note, however, that even when different exposure histories end up with the same cumulative exposure history; generally, they will not share the same probability of treatment and, consequently the same IPTW. In this way we estimate a composite (weighted average) effect of all the different observed histories that have the same cumulative exposure history. It is important to keep in mind that the resources transferred to the states by the federal government to meet the demands of the newly entitled were directly proportional to the number of people affiliated to the program. In this context, attaining a degree of coverage early in the exposure history of a SJ would represent roughly the same financial resources as attaining it at any other point in the future, but for a longer period.

Also, to draw on a larger pool of information, we assume that the DRFs were the same for all time periods, which allows us to pool across years including all 2328 SJ-year observations. Regarding standard errors and confidence intervals, the most straightforward way to estimate them is to bootstrap the entire estimation procedure, including the weights. Here we resample the set of of SJs and their histories, not the single SJ-year observations.

**Fig. 3:**
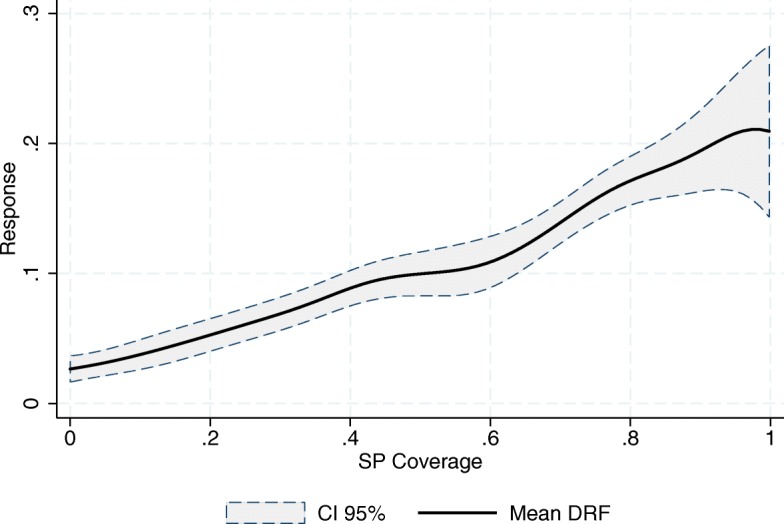
Increment in the Relative Number of Doctors’ Offices (per 1000 people outside the social security network). Source: Own elaboration based on data from the National Health Information System (SINAIS) and the administrative records of Seguro Popular

**Fig. 4:**
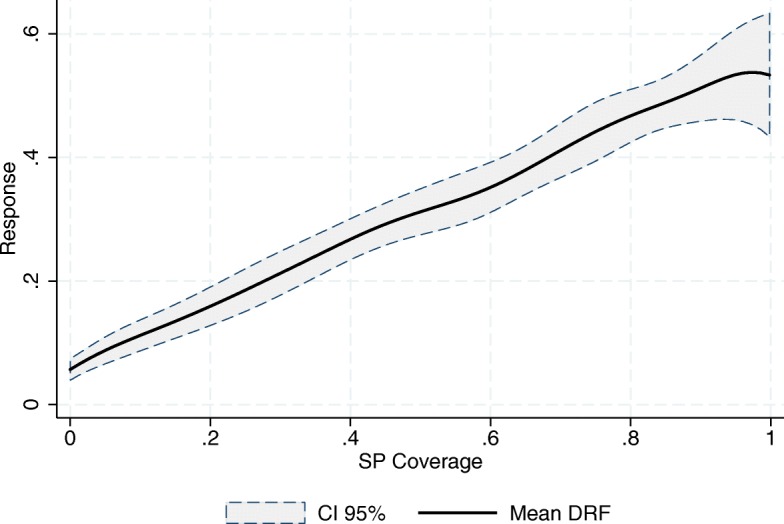
Increment in the Relative Number of Physicians in day-to-day Contact with Patients (per 1000 people outside the social security network). Source: Own elaboration based on data from the National Health Information System (SINAIS) and the administrative records of Seguro Popular

**Fig. 5:**
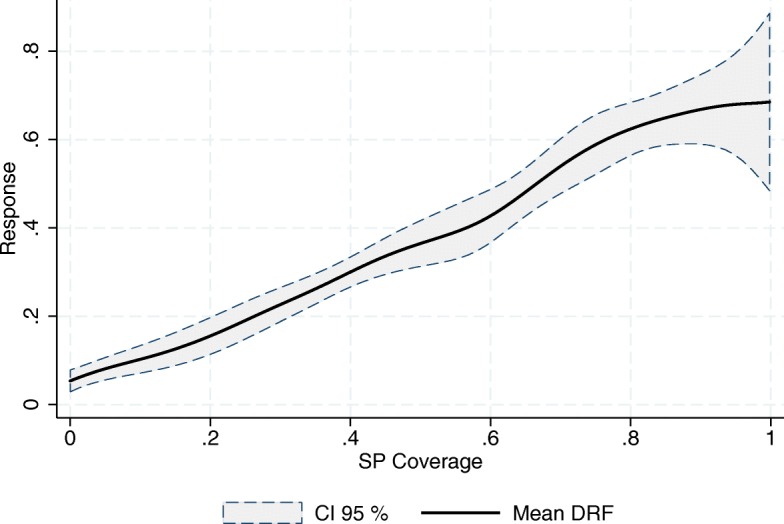
Increment in the Relative Number of Nurses in day-to-day Contact with Patients (per 1000 people outside the social security network). Source: Own elaboration based on data from the National Health Information System (SINAIS) and the administrative records of Seguro Popular

As mentioned earlier, we follow FFGN in estimating the DRFs using a kernel-weighted local linear regression of the outcome, *Y*_*t*_, on the cumulative exposure, $cum\left (\underline {A}_{k}\right)$, using the pseudo-sample generated by $\widehat {SW}_{t}\left (\underline {A}_{t},\underline {\mathbf {X}}_{t}\right)$. This is equivalent to multiplying each SJ-year observation’s kernel weight in the local linear regression by its corresponding $\widehat {SW}_{t}\left (\underline {A}_{t},\underline {\mathbf {X}}_{t}\right)$. Figures 3, 4 and 5 show the resulting mean DRFs for the observed increment in the relative number of SSA’s doctors’ doctors’ offices, physicians and nurses in day-to-day contact with patients, with respect to 2001, the year prior to the introduction of the program in its pilot phase. Note that, while $\underline {\mathbf {X}}_{t}$ are stocks (the density of resources), the outcomes are flows (increments).

The first thing to note in these graphs is that all three response curves exhibit a positive slope, meaning that the SP program has had, on average, a positive impact on the relative numbers of these human and material resources. Hence, we record an expansion in the provision of healthcare services. This result is in line with that of Bosch and Campos-Vázquez [6] and [7].

Also of importance is the difference between the end points of the mean response curves, *M* *e* *a* *n* *D* *R* *F*(1)−*M* *e* *a* *n* *D* *R* *F*(0), that is, the difference between full coverage and absence of SP affiliates. Our estimates suggest that, on average, the full coverage of the SP program would represent, for the SJs in our sample, an increase in the relative number of doctors’ offices, physicians and nurses providing clinical care (per 1000 people outside the social security network) of 0.18, 0.47 and 0.64 respectively. Given that in 2001 the densities of these resources were 0.45, 0.81 and 1 respectively, we can see that the program has had an important impact on the human and material resources of the SSA^9^.

The almost linear relationship depicted in all three graphs also suggests the absence of any economies of scale in the use of the financial resources being provided by the program. This is most striking in the case of doctors’ offices, where one would expect new infrastructure to be costlier. According to our estimates, the increment in the relative number of resources is, on average, directly proportional to the per-affiliate amount of money destined for the program.

A number to keep in mind is that in 2014 the government contribution reached an average of 2843.40 pesos (about 185 USD of the time) per affiliate, which constitutes 99% of the financial resources of the program ([32], p. 88). It is not hard to go from here to an estimate of how much it would cost to bring the relative number of resources to a desired level if things keep running the way they have been for the last decade. For example, in our sample of 194 SJs, in 2013 the mean relative number (per 1000 people outside the social security network) of SSA’ s physicians and nurses in day-to-day contact with patients – providing clinical care– was 1.2 and 1.6 respectively. Had the government contribution been 50% higher by that year, according to our estimates we would have seen an average density of physicians and nurses providing clinical care of 1.4 and 1.9, respectively.

### Heterogeneous effects: quantile dose-response functions

**Fig. 6:**
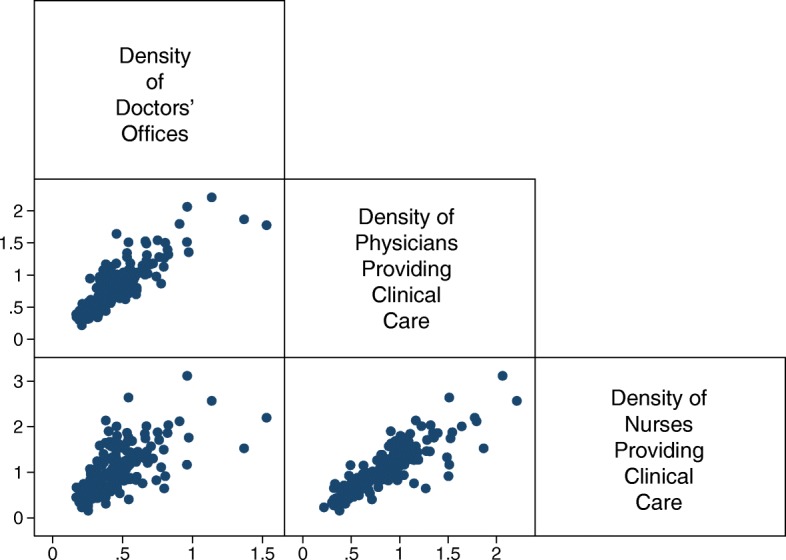
Relative Number of Human and Material Resources 2001 (per 1000 people outside the social security network). Source: Own elaboration based on data from the National Health Information System (SINAIS) and the administrative records of Seguro Popular. The numbers correspond to the 194 Sanitary Jurisdictions in common support

However informative the mean dose-response functions are, they do not provide a complete description of the program’s impact on other parts of the distribution of our outcome variables. It would be important to investigate the conditions under which the same coverage of the program has a differential response, possibly ascertaining subpopulations for which the program is most effective and help improve its design and steer it in the desired direction. We can see the relevance of this exercise in Fig. 6, that shows the differences in the densities of the resources in the 194 SJs in 2001 (the year prior to the introduction of the program). Observing this variability, heterogeneous responses to the program could be expected, even more so if what we observe in 2001 is the result of historical or natural obstacles that the SJs face to translate the financial resources into additional investments in personnel and infrastructure.

As previously stated, we can estimate quantile versions of the DRFs using the same kernel-weighted regression framework, only this time with linear quantile regressions of the outcome on the cumulative exposure. This will give us a clear understanding of how the program is affecting the whole distribution of our outcome variables, taking us one step further into characterizing the determinants of possible heterogeneous responses to the program.

**Fig. 7:**
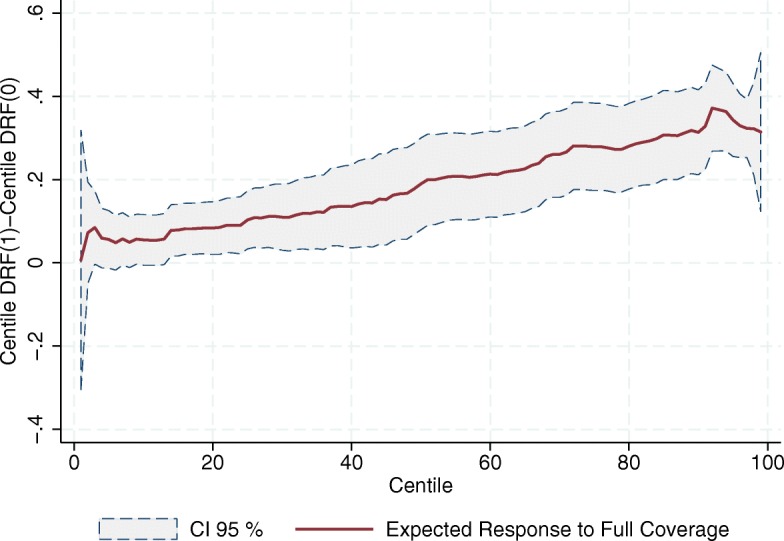
Expected Increment in the Relative Number of Doctors’ Offices due to Full Coverage of SP (per 1000 people outside the social security network). Source: Own elaboration based on data from the National Health Information System (SINAIS) and the administrative records of Seguro Popular

**Fig. 8:**
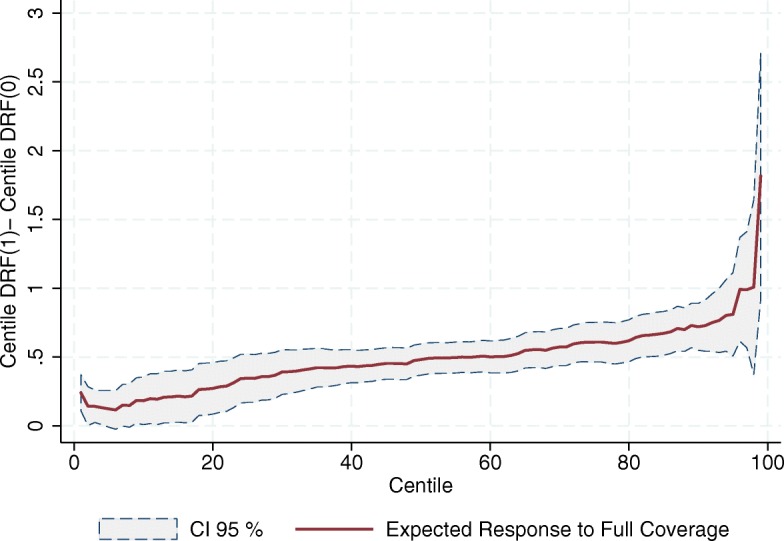
Expected Increment in the Relative Number of Physicians in day-to-day Contact with Patients due to Full Coverage of SP (per 1000 people outside the social security network). Source: Own elaboration based on data from the National Health Information System (SINAIS) and the administrative records of Seguro Popular

**Fig. 9:**
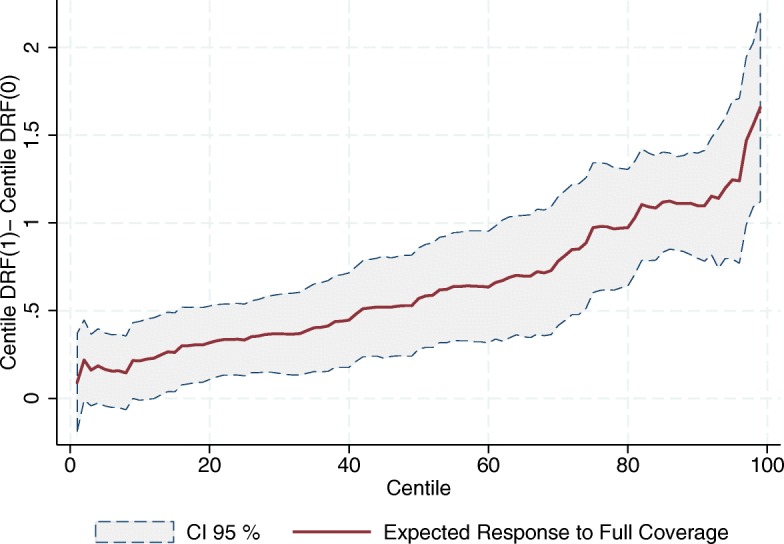
Expected Increment in the Relative Number of Nurses in day-to-day Contact with Patients due to Full Coverage of SP (per 1000 people outside the social security network). Source: Own elaboration based on data from the National Health Information System (SINAIS) and the administrative records of Seguro Popular

Figures 7, 8 and 9 show the estimated response to full coverage of SP on every centile of the distributions of the outcome variables; that is, the vertical difference between the end points of every centile *i* response curve, *C* *e* *n* *t* *i* *l* *e*_*i*_ *D* *R* *F*(1)−*C* *e* *n* *t* *i* *l* *e*_*i*_ *D* *R* *F*(0). All three figures show a positive tendency for the expected response to full coverage of the program. The fact that these figures do not exhibit horizontal lines suggests that going from zero to full coverage of SP has different responses along the distribution of the outcome variables, showing greater effects on the upper part of the distributions. In other words, the better the SJ is doing in seeing increased the relative number of its human and material resources, the greater its response to the program. Note that this is not a tautology; what this means is that the program gives greater boosts to those SJs increasing the most their resources – again almost in a direct proportion.

There are at least two reasons why this might be happening. The first is that it could be a simple reflection of the SJ’s investment priorities. It may well be that those SJs exhibiting greater increases in the relative number of their resources prioritized such expansion, and the program’s financial resources help them to do exactly that. If this were the case, it is only natural to observe that those SJs whose investment priorities lie elsewhere, other than expanding the density of their human and material resources, do not use the program’s money in this fashion.

A second reason for the positive slope in Figs. 7, 8 and 9 is that it might be reflecting historical obstacles the SJs face to transform financial means into human and material resources, obstacles that the program by itself cannot overcome. In this scenario, we would be looking not so much at the result of different investment priorities, but at technical difficulties in transforming the program’s budget into medical staff and infrastructure. It is well-known, for example, that it is costlier to invest in some areas due to limited geographical access. This alone would naturally lead to weaker responses to the program in in SJs where the need for resources is dearer.

**Fig. 10:**
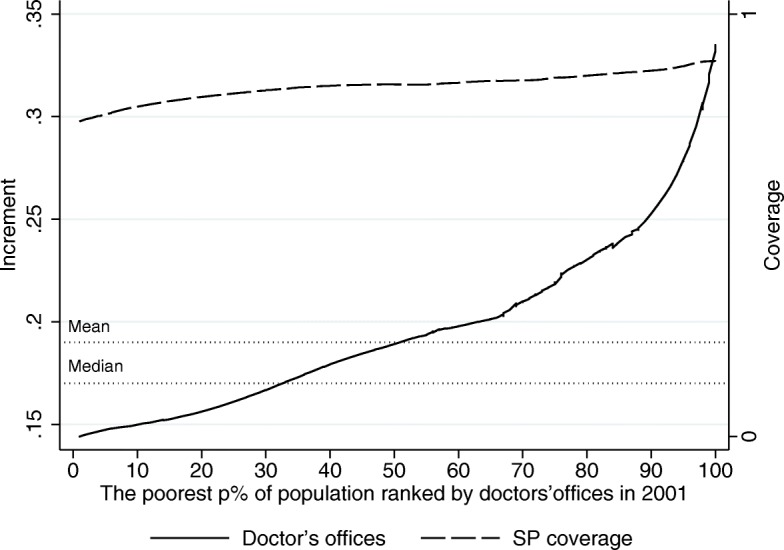
Increment Incidence Curve for the Relative Number of Doctors’ Offices, 2001–2013 (per 1000 people outside the social security network). Source: Own elaboration based on data from the National Health Information System (SINAIS) and the administrative records of Seguro Popular. Note: The horizontal lines depict the mean and median increment observed in the trimmed sample

**Fig. 11:**
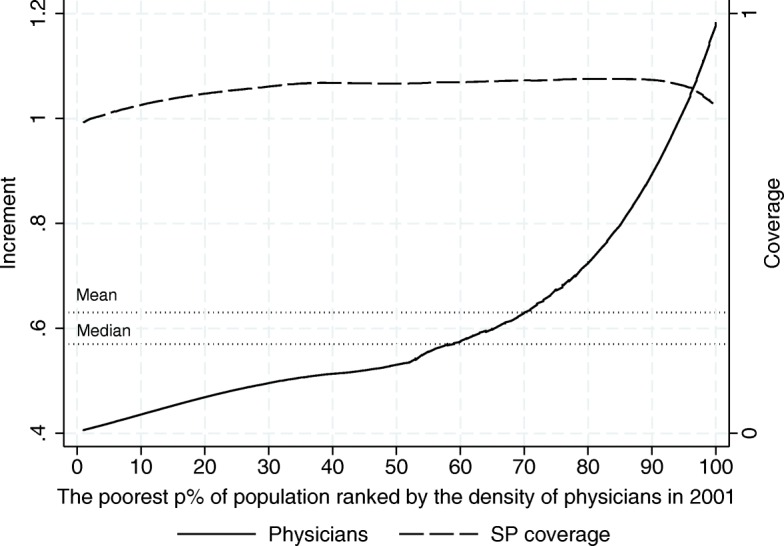
Increment Incidence Curve for the Relative Number of Physicians in day-to-day Contact with Patients, 2001–2013 (per 1000 people outside the social security network). Source: Own elaboration based on data from the National Health Information System (SINAIS) and the administrative records of Seguro Popular. Note: The horizontal lines depict the mean and median increment observed in the trimmed sample

**Fig. 12:**
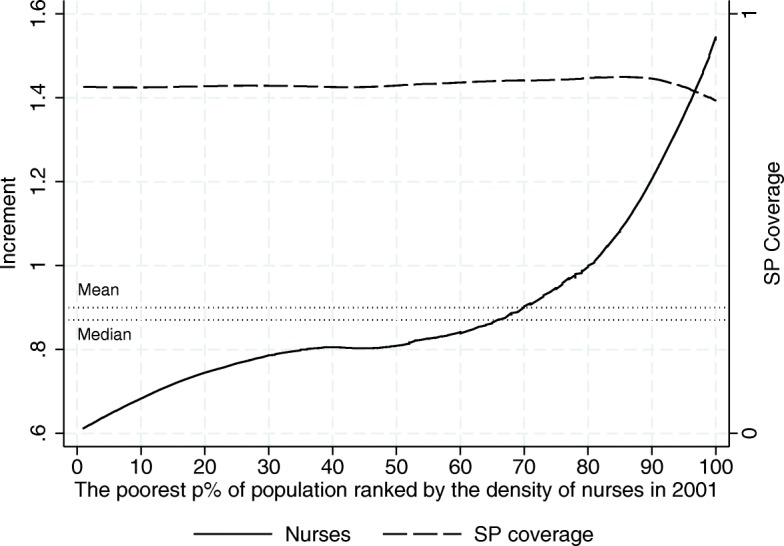
Increment Incidence Curve for the Relative Number of Nurses in day-to-day Contact with Patients, 2001–2013 (per 1000 people outside the social security network). Source: Own elaboration based on data from the National Health Information System (SINAIS) and the administrative records of Seguro Popular. Note: The horizontal lines depict the mean and median increment observed in the trimmed sample

In light of Figs. 7, 8 and 9, given that SP seems to have distributional effects on the outcome variables, it becomes relevant to determine where SJs fall in the distribution of our outcome variables, since this will tell us which SJs are making the most of the program in terms of the availability of resources needed in the provision of healthcare services. As an exploratory analysis we take a look at how the 2001–2013 increments in human and material resources were distributed across SJs according to their initial level^10^. In particular, Figs. 10, 11 and 12 show to what extent it can be said that the increment in these resources has been pro-poor in terms of the resource in question, reaching the most vulnerable parts of the population. As can be seen from this set of figures, all of our estimates suggest that the greatest increments in the relative number of human and material resources from 2001 to 2013, corresponded to those SJs that were better off in 2001 (before the pilot phase of the program). For example, where the average increment in the relative number of doctors’ offices in the initially poorer half of the preprogram distribution is between 0.14 and 0.19, in the upper half the increment ranges from 0.19 to 0.32 (Fig. 10).

Similar patterns are found for physicians and nurses in day-to-day contact with patients. We have also plotted the average SP coverage by decile in 2013 to show that this pattern is hardly explained by differences in the roll-out of the program. Nearly all the the SJs achieved complete program roll-out by 2013, but the eventual level of the outcome measure –the increment between 2001 and 2013– is almost entirely determined by where they were in 2001. Moreover, the dotted lines showing the mean and median increments observed in the trimmed sample demonstrate how limited the conclusion would be if we had only looked at the average treatment effect disregarding the huge differences in the impact the program has had across the SJs.

Perhaps as a natural result of the better-off SJs making the most of such a sizable program as SP, Fig. 13 shows the evolution of the absolute GINI^11^ for the material and human resources of the SSA over the 12-year period. There we can see that the inequality in the availability of resources has increased over time, implying that those SJs that were better off initially also expanded their resources at a faster pace, as suggested by Figs. 10, 11 and 12.

Together, the increment incidence curves and inequality indices suggest that the SJs making the least of the SP program are precisely those in greater need. The initially worse-off SJs are also the ones showing the least increment in basic human and material resources at roughly the same coverage levels of the program. Without making any assumption as to the degree in which SP has contributed to the more unequal distribution of health resources in Mexico, at this point, all we can say is that it has contributed. This should be a matter of major concern for any program directed at vulnerable population groups.

## Conclusions

We have analyzed the effects the implementation of a universal health insurance program in Mexico, Seguro Popular (SP), has had on key variables associated with the provision of healthcare services by the Secretaría de Salud –Ministry of Health– (SSA). The roll-out of SP unfolded over a period of more than a decade, with different treatment intensities across recipients –the Sanitary Jurisdictions (SJs) that are responsible for the provision of services. The expansion of insurance coverage and of the healthcare services formed a complicated interactive process that the milieu itself helped to create as it unfolded. Hence, the application of a traditional single-shot causal inference method would have implied imposing assumptions that do not reflect reality.

To control for the time-dependent confounding inherent to the dynamic nature of these kinds of programs, in this article we have brought together two strands of the literature on causal inference in observational studies: one focusing on fixed non-binary treatments and the other on binary dynamic treatments. The procedure we present here elaborates on Robins et al. [4] showing how to estimate mean and quantile dose-response functions of continuous dynamic treatments, much in the same fashion as Flores et al. [12] did for continuous fixed treatments; that is, applying local regression methods to appropriately weighted samples.

Our empirical set-up allows us to identify that SP has had a positive effect on the density of human and material resources available for healthcare services. We have shown that these positive effects grow in magnitude as we move to the right in the distribution of outcomes –that is, SJs with a stronger growth benefited more. Moreover, we have shown that those SJs that were better off in 2001 –before the roll-out of SP started– are the ones on the right of the outcome distribution showing the greater increases. Hence, unlike previous evaluations, we have found compelling quantitative evidence that the program has proven most helpful in less vulnerable territories, leaving behind those in greater need^12^.

Beyond the problems inherent to the lack of accountability [17], it is a well-known fact that rural and urban areas present quite different challenges for the provision of health services. In this sense, our findings might partly be due to efficiency concerns in the investment decisions at the state level. States in Mexico are faced with hard choices about the allocation and distribution of scarce resources and healthcare services. Firstly, input prices can vary substantially between rural and urban areas, presenting quite different challenges for the allocation. Secondly, given increasing returns to scale, it may well be costlier to deliver the same primary healthcare services in rural then in urban areas. Due to unequal geographical access, rural areas may not secure the economies of scale that urban areas achieve, making investment in the latter seem in line with cost-effectiveness considerations [33]. However, concerns with equity are also an important element of programs aimed at improving social conditions, and the pursuit of efficiency must never eclipse equity considerations in this context.

Even though Seguro Popular’s general aim of reducing inequalities appears uncontroversial, the practical notions of equity that should inform policy and the ways in which these should be implemented are far from clear. Seguro Popular does give absolute priority to those outside the social security network, a group previously without access to affordable healthcare services. However, our results indicate that the worst-off individuals among those are still left behind. In Mexico, there is an undeniable social gradient in health resources, meaning that the poorest areas are also those with the least healthcare resources. Also, it is well-known that poor people are more likely to have unhealthy lifestyles due to deficient educational services, local economic conditions, inadequate social support systems, and transportation. There is still a lot to be done in the quest for ensuring that, as far as possible, Mexican people have equal access to basic health resources. Without explicit considerations regarding a more specific priority setting, the situation depicted in this article is likely to remain unchanged; at least, our results indicate that the poorest regions have not been able to catch up. Moreover, in targeting the poorest of the poor, it may well be necessary to recognize that remote rural communities in Mexico can only receive primary healthcare through mobile medical units or telemedicine –and that even in that case, improved road infrastructure will be needed to guarantee access.

If Seguro Popular is taking efficiency to be paramount when distributing benefits, this gives rise to profound ethical concerns about the actual provision and financing of healthcare. Although perfect equality may not be achievable, whatever the trade-off between equity and efficiency in the allocation of healthcare resources, there should be an open discussion about the most cost-efficient ways of rationing those resources that also takes equity criteria into account.

## Appendix

**Table 2:** Descriptive Statistics of the Complete Sample^a^

Variable		Mean	Std. Dev.	Min	Max	Observations
Exposure	Overall	0.08	0.10	-0.39	0.77	*N*= 2796
	Between		0.01	0.03	0.12	*n*=233
	Within		0.10	-0.40	0.76	*T*= 12
Cumulative exposure	Overall	0.39	0.34	0.00	1.27	*N*= 2796
	Between		0.14	0.08	0.89	*n*=233
	Within		0.31	-0.50	1.13	*T*= 12
Doctors’ offices	Overall	0.59	0.37	0.15	3.25	*N*= 2796
	Between		0.36	0.16	3.02	*n*=233
	Within		0.09	-0.07	1.98	*T*= 12
Staffed hospital beds	Overall	0.65	0.86	0.00	9.70	*N*= 2796
	Between		0.85	0.00	6.57	*n*=233
	Within		0.18	-1.72	4.31	*T*= 12
Non-staffed hospital beds	Overall	0.60	0.47	0.00	5.18	*N*= 2796
	Between		0.38	0.06	3.55	*n*=233
	Within		0.27	-0.63	3.04	*T*= 12
Physicians in day-to-day contact with patients	Overall	1.28	1.13	0.22	11.38	*N*= 2796
	Between		1.10	0.30	10.51	*n*=233
	Within		0.25	-1.06	3.75	*T*= 12
Physicians without day-to-day contact with patients	Overall	0.10	0.17	0.00	2.22	*N*= 2796
	Between		0.16	0.01	1.52	*n*=233
	Within		0.05	-0.49	1.18	*T*= 12
Nurses in day-to-day contact with patients	Overall	1.65	1.60	0.11	17.03	*N*= 2796
	Between		1.57	0.28	14.29	*n*=233
	Within		0.32	-1.20	4.38	*T*= 12
Nurses without day-to-day contact with patients	Overall	0.10	0.14	0.00	1.35	*N*= 2796
	Between		0.12	0.00	1.14	*n*=233
	Within		0.06	-0.35	1.00	*T*= 12

Source: Own elaboration based on data from the National Health Information System (SINAIS) and the administrative records of Seguro Popular

^a^The table shows the standard deviation decomposed into between $\left (\overline {x}_{i}\right)$ and within $\left (x_{it}-\overline {x}_{i}+\overline {\overline {x}}\right)$ components

**Table 3:** Descriptive Statistics of the Trimmed Sample^a^

Variable		Mean	Std. Dev.	Min	Max	Observations
Exposure	Overall	0.08	0.09	-0.20	0.67	*N*= 2328
	Between		0.01	0.05	0.12	*n*=194
	Within		0.09	-0.21	0.67	*T*= 12
Cumulative exposure	Overall	0.38	0.33	0.00	1.21	*N*= 2328
	Between		0.12	0.15	0.77	*n*=194
	Within		0.31	-0.39	1.12	*T*= 12
Doctors’ offices	Overall	0.50	0.22	0.15	1.81	*N*= 2328
	Between		0.20	0.16	1.56	*n*=194
	Within		0.09	-0.17	1.89	*T*= 12
Staffed hospital beds	Overall	0.43	0.26	0.00	2.16	*N*= 2328
	Between		0.24	0.00	1.39	*n*=194
	Within		0.09	-0.09	1.19	*T*= 12
Non-staffed hospital beds	Overall	0.52	0.34	0.00	2.44	*N*= 2328
	Between		0.26	0.06	1.43	*n*=194
	Within		0.23	-0.54	1.60	*T*= 12
Physicians in day-to-day contact with patients	Overall	1.00	0.41	0.22	2.71	*N*= 2328
	Between		0.37	0.30	2.15	*n*=194
	Within		0.19	0.14	2.04	*T*= 12
Physicians without day-to-day contact with patients	Overall	0.07	0.05	0.00	0.30	*N*= 2328
	Between		0.04	0.01	0.22	*n*=194
	Within		0.03	-0.02	0.27	*T*= 12
Nurses in day-to-day contact with patients	Overall	1.24	0.55	0.11	3.49	*N*= 2328
	Between		0.48	0.28	2.80	*n*=194
	Within		0.26	0.29	2.36	*T*= 12
Nurses without day-to-day contact with patients	Overall	0.07	0.06	0.00	0.54	*N*= 2328
	Between		0.05	0.00	0.23	*n*=194
	Within		0.03	-0.11	0.38	*T*= 12

Source: Own elaboration based on data from the National Health Information System (SINAIS) and the administrative records of Seguro Popular

^a^The table shows the standard deviation decomposed into between $\left (\overline {x}_{i}\right)$ and within $\left (x_{it}-\overline {x}_{i}+\overline {\overline {x}}\right)$ components

## Abbreviations

ATE: Average treatment effect
CBPS Covariate balancing propensity score; CONAPO: Consejo Nacional de Población
DGP: Data-generating process
DRF: Dose-response function
GLM: Generalized linear model
GM: Genetic matching
GMM: Generalized method of moments
GPS: Generalized propensity score
IMSS: Intituto Mexicano del Seguro Social
IPTW: Inverse probability of treatment weights
IPW: Inverse probability weights
ISSSTE: Instituto de Seguridad y Servicios Sociales de los Trabajadores del Estado
LGS: Ley General de Salud –General Health Law–
MSM: Marginal structural model
OLS: Ordinary least squares
PEMEX: Petróleos Mexicanos
PS: Propensity score
QTE: Quantile treatment effect
REPSS: Regímenes Estatales para la Protección Social en Salud –State Regimes for Social Protection in Health–
RMSE: Root mean squared error
SINAIS: Sistema Nacional de Información en Salud –National Health Information System–
SJ: Sanitary jurisdiction
SP: Seguro popular
SPSS: Sistema de Protección Social en Salud –system of social protection in health–
SSA: Secretaría de Salud –ministry of health–
TSCS: Time-series cross-sectional
UNEMES: Unidades de Especialidades Médicas

## Footnotes

1 Nearly all treatments of epidemiological interest present these characteristics –imagine a drug whose dose is readjusted according to the patient’s clinical response. Recently these tools, originally developed in biostatistics and epidemiology, have been applied in political science [8] to study the effectiveness of a candidate’s decision to “go negative” in political campaigns, an inherently dynamic process.

2 Our exposure variable is the (continuous) treatment intensity, that is, the number of SP affiliates relative to the number of eligible people, in each Sanitary Jurisdiction. The latter, therefore, are our program recipients.

3 FFGN proposed to estimate the mean DRF of a fixed continuous intervention using a kernel-weighted local linear regression of the outcome, *Y*, on the exposure, *A*, where each recipient’s kernel weight is divided by its exposure assignment model pdf properly evaluated.

4 The data is available at a yearly frequency disaggregated at the municipality level and can be retrieved from the SINAIS web site: http://www.dgis.salud.gob.mx/contenidos/sinais/estadisticas.html.

5 See http://dgces.salud.gob.mx/INDICASII/.

6 It is important to note that it was not necessarily the case that every SJ got their corresponding share of financial resources, because the Regímenes Estatales para la Protección Social en Salud –State Regimes for Social Protection in Health– (REPSS) determined how and where to invest the program’s financial resources.

7 Since the SJs are comprised of several municipalities, we use the census data to approach the size of the target population.

8 Physicians and nurses without day-to-day contact with patients include those engaged in administrative tasks, educational activities, epidemiologists, and anatomic-pathologists.

9 The World Health Organization considers less than 2.3 health workers providing clinical care per 1000 people as a critical shortage.

10 A thorough investigation into the distributional effects would require the development of new procedures to estimate counterfactual distributions under different possible scenarios, a pending assignment for time-varying continuous interventions.

11 The absolute GINI inequality index, advocated by Kolm [34, 35], is unaffected by an equal addition to all incomes (translation invariant). while in the standard (relative) GINI index inequality is unchanged when all incomes are increased in the same proportion (scale invariant). Several other indices were also estimated showing the same tendency. Given that the absolute GINI index, that always considers improvement in terms of absolute difference, is not unit invariant we have normalized all indices so that 2002=100.

12 If, for example, we had ignored the dynamic nature of our empirical application, using only preprogram covariates in an attempt to control for confounders, evidence suggests that we would have overestimated the mean impact of the program [7], as we would have missed the fact that those SJs better off were the same making the most of the program at every point in time.
